# Detection of Vancomycin Resistance among Methicillin-Resistant *Staphylococcus aureus* Strains Recovered from Children with Invasive Diseases in a Reference Pediatric Hospital

**DOI:** 10.3390/antibiotics13040298

**Published:** 2024-03-26

**Authors:** Lorena Pardo, María Inés Mota, Andrés Parnizari, Adriana Varela, Gabriela Algorta, Gustavo Varela

**Affiliations:** 1 Bacteriology and Virology Academic Unit, Facultad de Medicina, Universidad de la República, Montevideo 11600, Uruguay; imota@higiene.edu.uy (M.I.M.); aparnizari@higiene.edu.uy (A.P.); 2 Pediatric “C” Academic Unit, Facultad de Medicina, Universidad de la República, Montevideo 11600, Uruguay; 3 Bacteriology Laboratory, “Pereira Rossell” Pediatric Hospital, Montevideo 11600, Uruguay; adrianavarela16@gmail.com (A.V.); algortagabriela@gmail.com (G.A.)

**Keywords:** *S. aureus*, vancomycin resistance, methicillin-resistant *Staphylococcus aureus*, hVISA

## Abstract

Vancomycin is the cornerstone in treating methicillin-resistant *Staphylococcus aureus* (MRSA) infections. However, therapeutic failures can occur when MRSA strains with decreased susceptibility to glycopeptides (DSG) are involved. The aim of this study was to detect and characterize DSG in MRSA recovered from children with invasive diseases at a reference pediatric hospital between 2009 and 2019. Fifty-two MRSA strains were screened using agar plates with vancomycin 3 and 4 mg/L (BHI-3 and BHI-4); the VITEK2 system; and standard and macro *E*-tests. Suspicious hVISA were studied by population analysis profiling–area under the curve (PAP-AUC), and wall thickness was analyzed by transmission electron microscopy. Neither VRSA nor VISA were detected in this set. As only three strains met the hVISA criteria, the PAP-AUC study included 12 additional MRSA strains that grew one colony on BHI-4 plates or showed minimum inhibitory concentrations of vancomycin and/or teicoplanin ≥ 1.5 mg/L. One strain was confirmed as hVISA by PAP-AUC. The wall thickness was greater than the vancomycin-susceptible control strain; it belonged to ST30 and carried SCC*mec* IV. As expected, a low frequency of hVISA was found (1.9%). The only hVISA confirmed by PAP-AUC was not detected by the screening methods, highlighting the challenge that its detection represents for microbiology laboratories.

## 1. Introduction

Vancomycin is the antibiotic of choice to treat methicillin-resistant *Staphylococcus aureus* (MRSA) infections in both children and adults, especially in severe diseases. This antibiotic inhibits cell wall formation by hiding peptidoglycan precursors, which is an important bacterial structure [1].

In 2001, Hiramatsu et al. described *S. aureus* strains with de novo resistance to vancomycin, a minimal inhibitory concentration (MIC) greater than 8 mg/L and reported them as vancomycin-resistant *Staphylococcus aureus* (VRSA), according to the British Society for Antimicrobial Chemotherapy guidelines. These strains showed a change in the cell wall precursor codified by the gene *van*A, as described in *Enterococcus* [2].

In 2006, the Clinical and Laboratory Standards Institute (CLSI) lowered the MIC value to define intermediate resistance or decreased susceptibility based on therapeutic failures in diseases caused by *S. aureus* with vancomycin MICs of 4 mg/L. *S. aureus* is currently classified as vancomycin-susceptible (VSSA), vancomycin-intermediate (VISA) and vancomycin-resistant (VRSA) according to the following MIC values: less than or equal to 2, between 4 and 8, and greater than 8 mg/L, respectively. Strains with decreased susceptibility due to MICs between 4 and 8 mg/L, so-called vancomycin-intermediate *Staphylococcus aureus* (VISA), have been described worldwide [3].

The expression of intermediate resistance can be homogeneous (VISA) or heterogeneous (hVISA); the latter phenotype makes its detection difficult by routine laboratory bacteriological procedures. Automated systems frequently used in clinical laboratories and disk diffusion tests are not recommended for the detection of these strains. Additionally, these strains are associated with treatment failures and persistent infections [3,4].

The genetic mechanisms responsible for the hVISA phenotype are partially known. There is no conclusive evidence that resistance is maintained over time once vancomycin is withdrawn nor that VISA strains can be transmitted to other people and cause disease [5].

Updated information about antibiotic resistance is important for the development or modification of therapeutic algorithms; on the other hand, analyses conducted over time and in different countries to recover more hVISA strains allow us to study them in detail with new laboratory procedures and also to understand the global phenomenon of resistance, the context in which it occurs and how it is established over time [6,7].

Most hVISA strains described from Asia, South America and Europe are MRSA isolated from adults and belong to ST5, ST8 and ST239. There are no data about the prevalence and microbiological characteristics of hVISA in MRSA recovered from children in Uruguay [6,7,8]. The aim of this research was to detect decreased susceptibility to glycopeptides (DSG) in MRSA strains isolated from children with invasive diseases assisted at the local reference public hospital between 2009 and 2019.

## 2. Results

**Figure 1:**
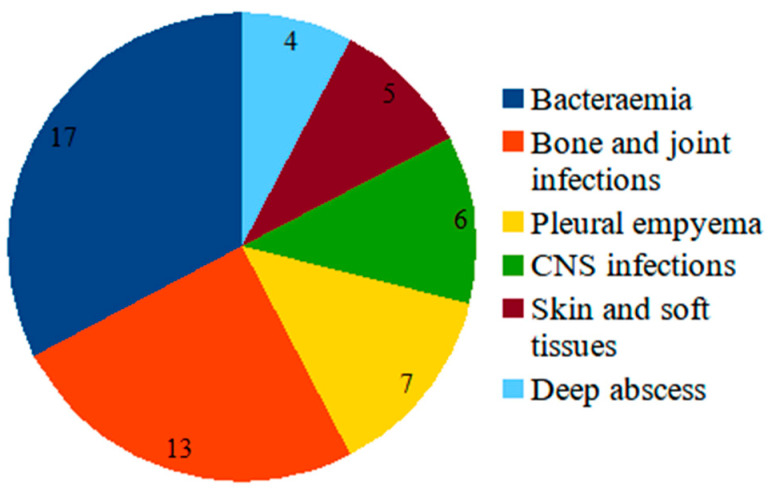
Distribution of the 52 MRSA strains according to the type of infection in Uruguay, 2009–2019. CNS, central nervous system.

Fifty-two MRSA strains from invasive diseases were recovered during the period 2009–2019. The majority corresponded to bacteremia (17) and bone and joint infections (13), which were followed by pleural empyema (7), central nervous system infections (6), skin and soft tissue infections (5), and deep abscesses (4) (Figure 1).

### 2.1. mecA, lukS-F Genes, SCCmec and MLST Genotyping

**Figure 2:**
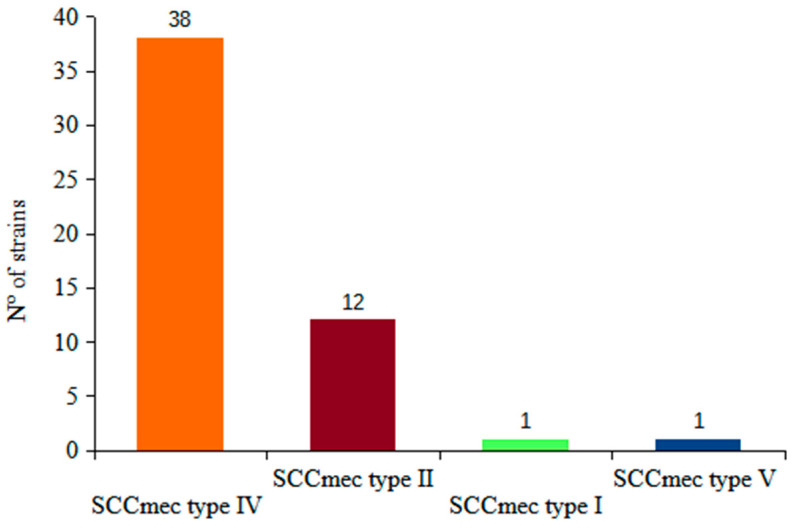
Distribution of the 52 MRSA strains according to SCC*mec* type, Uruguay 2009–2019.

All 52 MRSA strains harbored *mec*A gene and 47 carried the *lukS-F* gene. The distribution of the SCC*mec* types was as follows: SCC*mec* type IV 38 strains; SCC*mec* type II 12 strains, and one isolate each belonged to type I and V (Figure 2). Details on the obtained sequence type (ST) and the clonal complex (CCs) of the 10 selected strains are provided below.

### 2.2. Antimicrobial Resistance Profile According to VITEK2 MICs Results

Thirteen strains exhibited resistance to macrolide–lincosamide–streptogramin B. Among these, four showed the inducible (iMLSb) phenotype, while in nine, the phenotype was constitutive (cMLSb). Resistance to ciprofloxacin (MIC ≥ 8 mg/L) was observed in seven strains, while there was resistance to erythromycin in 3 (MIC ≥ 8 mg/L), to gentamicin (MIC ≥ 16 mg/L) in 1, and to trimethoprim–sulfamethoxazole in 1 (MIC ≥ 16 mg/L).

### 2.3. Screening for Glycopeptide Resistance and Population Analysis Profile–Area under the Curve (PAP-AUC) Assay

Both, vancomycin and teicoplanin for MIC_90_ were 1.5 mg/L using the standard *E*-test.

No MRSA strains exhibited a MIC exceeding 4 mg/L for vancomycin or 8 mg/L for teicoplanin, as determined by either the standard or macro *E*-test. None of the 52 MRSA isolates displayed glycopeptide susceptibility reduced (GSR) based on MIC results obtained by Vitek2 (BioMerieux, Marcy-l’Étoile, France), *E*-test, and macro *E*-test.

Using brain–heart infusion agar supplemented with 3 mg/L of vancomycin (BHI-3), 36 strains (69%) grew after 48 h of incubation.

Three strains met the defined criteria (the presence of two or more colonies) on BHI-4 agar, while eight strains developed only a single colony by the end of the incubation period.

The BHI-3 results were excluded from further analysis due to the high number of positive results (36 out of 52 strains). The cut-off value in BHI-3 may be low for effective screening. Since only three strains (#2, #10 and #14) from Table 1 met the previously described hVISA criteria (growth of two or more colonies from a single drop after 24 or 48 h on BHI-4 plates), we decided to include additional strains for the PAP-AUC assay. This additional criteria included either a count of 1 CFU per drop after 48 h of incubation on BHI-4 plates or an MIC ≥ 1.5 mg/L to vancomycin or teicoplanin by standard *E-*test (n = 12) (Table 1).

A total 15 out of the 52 MRSA strains met the modified criteria to be studied by the PAP-AUC assay (Table 1).

Only one (#7) of these fifteen strains meeting the hVISA criteria showed an AUC/AUCμ3 ratio value greater than 0.9 (Table 1). This strain had MIC values of 1 and 0.5 mg/L for vancomycin and teicoplanin, respectively, as determined by Vitek2. In the macro *E*-test, it showed values of 2 mg/L for vancomycin and 1.5 mg/L for teicoplanin. No growth was observed on BHI4 at 24 h, but a single colony developed after 48 h of incubation. This strain was recovered from blood cultures obtained from a child with a catheter-related bloodstream infection.

Results for the remaining 14 strains, including the AUC, AUC/ AUCμ3 ratio, vancomycin and teicoplanin MICs (by Vitek, *E*-test, Macro *E*-test) and screening test using BHI-4, are shown in Table 1.

### 2.4. Microbiological Characteristics of the Suspect hVISA Strains

As detailed in Table 2, 15 MRSA strains selected as potential hVISA displayed various microbiological characteristics, including the methicillin-associated resistance phenotype and infection sites. Five strains were recovered from children with bacteremia (included strain #7), along with four from arthritis, two from pneumonia, two from osteomyelitis, one from endocarditis and one from adenophlegmon (Figure 1).

Distribution according to the SCC*mec* type was as follows: type II, four strains; type IV, nine strains; and type V, two strains. MLST analysis was performed on 10 of these strains, of which three belonged to ST-30, two to ST-5, two to ST-8, and one each to ST-868, 434 and 577. Six strains carried the *lukS-F* gene (Figure 2).

The strain with an AUC/AUCμ3 ratio greater than 0.9 carried the *lukS-F* gene, SSC*mec* type IV and belonged to ST-30.

### 2.5. Analysis of Cell Wall Thickening

**Figure 3:**
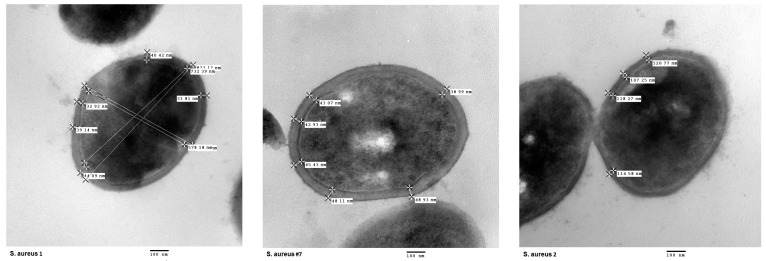
Cell wall thickness of the 3 studied strains by transmission electron microscopy. From left to right, *S. aureus* 1: ATCC 259213 (vancomycin-susceptible control strain); hVISA #7: strain (recovered in this study); and *S. aureus* 2: VISA strain Mu50 ATCC 700699 (VISA control strain). The lines indicate the sector of the cell wall that was measured, and the labels show the measurement value in nm.

Cell wall thickness in the presence of vancomycin was measured from the single strain with the PAP-AUC value of 0.96 (#7, see Table 1 and Table 2), which showed an average of 37.81 SD 5.47. This value was higher than the measurement of the vancomycin-susceptible control strain ATCC 259213 (31.63 SD 4.49) and lower than the VISA control strain Mu ATCC 700699(51.40 SD 9.17). The difference between these three strains was significant (*p* = 0.001). Figure 3 shows the images obtained by transmission electron microscopy.

## 3. Discussion

This work aimed to detect MRSA isolates with DSG recovered from samples of invasive pediatric infectious disease. The strategy involved analyzing a collection of 52 MRSA strains obtained between 2009 and 2019, using different previously described screening methods. Because most reports suggest that the frequency of hVISA, although increasing, is low and related to the use of vancomycin, we proposed to analyze all MRSA strains isolated over an extended period in a reference pediatric hospital which serves children from all over the country and where the use of vancomycin has increased since 2002. In doing so, we may be able to increase the likelihood of recovering strains with DSG to glycopeptides [7,8].

This study did not evaluate the effectiveness of glycopeptide or other antibiotic treatments during the analyzed period.

Among this set of 52 MRSA isolates, no homogenous VISA or VRSA strains were found. This result was consistent with the surveillance of DSG in the world. Globally, the frequency of VRSA is close to 1% and has been increasing in recent years, reaching 2.4% of *S. aureus* isolates. Conversely, the occurrence of VISA and hVISA is slightly higher, at 4.3% and 5.3%, respectively [7]. In this study, only three strains (6%) tested positive according to classical interpretive criteria for the used methods (see Table 1), but none of them were confirmed by PAP-AUC assay (see Table 2). The hVISA isolate identified in this study was obtained in 2014 from a child with catheter-related bacteremia, underlying comorbidities, and a prolonged hospitalization with extensive antibiotic exposure, including vancomycin.

While there are descriptions of hVISA in methicillin-susceptible *Staphylococcus aureus* (MSSA), most were detected in MRSA strains and associated with multidrug-resistant phenotypes. However, the hVISA strain #7 was SCC*mec* IV and did not show a multidrug-resistant phenotype. On the other hand, in the neighboring countries Argentina and Brazil, there are descriptions of hVISA strains in both multidrug-resistant and non-multidrug-resistant MRSA phenotypes [9,10,11].

The hVISA phenotype is commonly reported in MRSA strains belonging to ST5, ST8 and ST239. However, the hVISA strain recovered in this research, corresponding to ST30, exhibited similar microbiological characteristics to an hVISA strain isolated from a neonate’s blood culture in Brazil in 2009 [10]. The ST30 (CC30) has been circulating in the region and Uruguay since the beginning of the burden of community-acquired MRSA (CA-MRSA) infections and remains present today [12,13,14]. Therefore, considering the proposed mechanisms for hVISA emergence, it is plausible that this specific ST had been exposed to vancomycin for an extended period, especially given the documented increase in vancomycin use in this hospital since the 2002 CA-MRSA outbreak in Uruguay [5,8]. It is unknown whether this child was infected with a hospital-successfully established hVISA-MRSA clone or whether it was derived from an originally susceptible strain selected by vancomycin exposure. In favor of the second hypothesis is the absence of hVISA isolation from other children. However, this could be due to the aforementioned difficulty in recovering hVISA. Anyway, to accept or reject these hypotheses, other studies that could recover and compare hVISA strains from the same patient obtained at different times, from different health care centers, and patients assisted in the same service, with more powerful genetic tools such as Whole Genome Sequencing methods are required [15].

The origin of the DSG remains debatable and would be related to changes in the homeostasis of the bacterial wall. These changes include insufficient cross-linking in the cell wall that entrap glycopeptides at the periphery; an increase in the synthesis of the cell wall through the increased addition of N-acetylglucosamine; and the release of cell wall precursors outside the bacteria. All of these mechanisms could prevent the antibiotic from reaching the site of action, eliminating susceptible bacteria, and selecting those with higher MICs than the original wild strains [2].

The mean cell wall thickness hVISA #7 treated with vancomycin was greater than the reference susceptible strain (ATCC 259213) but less than the reference VISA strain (Mu ATCC 700699). Some authors describe the heterogeneous susceptibility as an intermediate step between sensitivity and resistance, which is probably due to the accumulation of changes in the genes that regulate wall synthesis [16].

The hVISA phenotype poses a significant challenge for laboratory detection and can potentially lead to treatment failure, especially in patients with comorbidities and prolonged exposure to vancomycin. The current methods for detecting hVISA have limitation, as evidenced by the difficulty in identifying these strains using the selected screening methods with the cut-off points proposed by the authors. Richter et al. found that 10.5% of isolates with a vancomycin MIC of 2 mg/L were hVISA compared to 0.1% of those with an MIC of 1 mg/L [17].

BHI-3 screening was not considered, since more than half of the isolates resulted positive. Isolates with borderline results from other screening methods (n = 15) were confirmed by PAP-AUC, which is the gold standard for hVISA detection. The threshold of 4 mg/L of vancomycin gives fewer false positive results than lower concentrations [18]. The BHI-V4 sensitivity for 2 or 1 CFU was 98.5% and 99.8%, respectively, but the specificity decreased from 93.8% to 89.8%, respectively [18]. In this study, results at 24 h failed to detect hVISA #7. Some authors describe hVISA strains as a “slow” phenotype, whose colonies appear later after being incubated with vancomycin, which could explain this result. These strains present alterations in genes involved in metabolic pathways such as purines, pyrimidine and peptidoglycan synthesis [19,20].

The Macro *E*-test method is not reliable for the detection of DSG with a sensitivity of 57% as described by Satola et al. In this work, hVISA #7 was not detected by this method [19].

The PAP-AUC study is a laborious laboratory technique, which cannot be performed routinely in most clinical bacteriology laboratories; thus, finding simple and reliable screening methods is relevant [21].

Although the detection of these strains remains difficult, the CDC proposes that laboratories that use automated methods should also search VISA and/or VRSA using BHI screening agar plates that contain 6 mg/L of vancomycin, noting that this method would only detect strains with MICs of 8 mg/L or higher [22]. There is no recommended method for hVISA screening.

## 4. Limitations

This study included MRSA strains isolated from a single health care center; however, the “Pereira Rossell” Pediatric Hospital (PRPH) is a pediatric reference hospital for medical and surgical specialties, and it receives children from all over the country. Further local investigations should include MRSA isolates recovered from adults, since worldwide, the majority of hVISA infections were described in that age group. Additionally, other laboratory methods, like pre-diffusion tablets, could have been used as screening procedures for strains with DSG [23]. 

## 5. Materials and Methods

A descriptive, retrospective study was carried out in PRPH, a tertiary-level public and teaching hospital which is a national reference center for all medical and surgical specialties. It provides high-level annual care to more than 300,000 children under 15 years old from all over the country, most of whom are from low-income homes.

### 5.1. Bacterial Strains

Only methicillin-resistant *S. aureus* (MRSA) isolates obtained from invasive disease (one per child) in the period from 1 January 2009 to 31 December 2019 were included in this study. Staphylococcal invasive disease was defined when *S. aureus* was recovered from a normally sterile body site: blood, bone, profound skin and soft tissue infections such as cellulitis or deep abscess, devices related to infections as well as from pleural, cerebrospinal and joint fluids. MRSA strains were identified by the Vitek2 system (BioMerieux, Marcy-l’Étoile, France). All analyzed MRSA were stored in skim milk at −20 °C for further analysis.

### 5.2. Search for mecA, lukS-F Genes, SCCmec and Multilocus Sequence Typing (MLST)

Bacterial DNA was obtained from isolated colonies after overnight incubation using the Wizard genomic DNA preparation kit (Promega, Madison, WI, USA) adding 20 mg/mL lysostaphin (Sigma Aldrich, St. Louis, MO, USA) in the cell lysis step.

Detection of the *mec*A gene was performed by PCR with primers MECA P4 and MECA P7 at a final concentration of 4 µM. The cycling conditions were as follows: initial denaturation at 94 °C for 5 min; 35 cycles of denaturation at 94 °C for 30 s, annealing at 53 °C for 30 s, and extension at 72 °C for 1 min, which was followed by a final extension at 72 °C for 5 min, as described by Oliveira et al. in 2002 [24].

SCC*mec* typing was performed according to a multiplex PCR assay described by Kondo et al. in 2007 [25]. This strategy aims to classify both the *ccr* gene complex (*ccr*) and *mec* gene complex (*mec*). The combination of these defines the SCC*mec* type. Details of the primers used, amplification conditions, and the interpretation algorithm are shown in Table 3 and Table 4.

A reaction mixture with a final volume of 25 μL per tube containing the following compounds was used: 0.4 μM of both primers, forward and reverse of the target sequence, 0.5 mM of dNTPs; 1 × PCR Buffer; 1.5 mM MgCl_2_; 0.04 U/μL of *Taq* polymerase (Bioline^®^, London, UK) and 50 ng of DNA template. To identify complex A, the amplification conditions were as follows: 94 °C for 5 min, followed by 30 cycles of 94 °C for 30 s, 52 °C for 1 min, and 72 °C for 1 min; with a final extension at 72 °C for 4 min. To identify complex B, the annealing temperature was 55 °C, and for complex C, *ccr*AB and *ccr*C were 50 °C. 

Once the types of *mec* and *ccr* complexes were identified, the following schemes (Table 4) were used to establish the SCC*mec* type.

The detection of *lukS-F* genes was performed also by PCR using the primers *lukS-F* at a final concentration of 2 µM and the following conditions: initial denaturation for 5 min at 94 °C; 35 cycles of denaturation for 40 s at 94 °C, annealing for 40 s at 55 °C, and extension for 1 min at 72 °C; and a final extension for 10 min at 72 °C, according to Lina in 1999 [27].

Multilocus Sequence Typing (MLST) was performed using the protocol by Enright et al. in 2000 on 10 of the 15 MRSA strains selected for PAP-AUC analysis (see above in Section 2). Briefly, PCR amplified 7 genes (*arcC*, *aroE*, *glp*, *gmk*, *pta*, *tpi*, and *yqiL)* using recommended primers under the following conditions: initial 5min denaturation at 95 °C, followed by 30 cycles of annealing at 55 °C for 1 min, extension at 72 °C for 1 min, and denaturation at 95 °C for 1 min, followed by a final extension step of 72 °C for 5 min. Amplicons were purified with a Wizard^®^ SV Geland PCR Clean-Up System (Promega Corporation, Madison, WI, USA) and sequenced at the Instituto Pasteur de Montevideo (Montevideo, Uruguay). Sequences were analyzed using the MLST website (https://pubmlst.org/ (accessed on 1 January 2023)) [28].

### 5.3. Antimicrobial Susceptibility Testing

Susceptibility testing for ciprofloxacin, gentamicin, trimethoprim–sulfamethoxazole, erythromycin, clindamycin, vancomycin and rifampin was performed using the VITEK2 system, and results were interpreted according to the standards of the 2023 Clinical & Laboratory Standards Institute (CLSI) guidelines [29].

### 5.4. Screening for Resistance and Decreased Susceptibility to Glycopeptides

Fresh cultures were obtained from the MRSA stored in skim milk at −20 °C. All strains were independently tested in duplicate to assess the reproducibility of each method. All the techniques were carried out on the same day and using the same inoculums.

#### 5.4.1. Standard *E*-Test

Vancomycin and teicoplanin MICs were determined following CLSI recommendations. ATCC *S. aureus* 29213, ATCC *E. faecalis* 29212 and 51299 were included as quality controls [29].

#### 5.4.2. Macro *E*-Test

The macro *E*-test was performed using a 2.0 McFarland inoculum standard on BHI agar plates (Difco, Becton Dickinson and Company, Franklin Lakes, NJ, USA) with commercial *E*-test strips containing vancomycin and teicoplanin. Resistance was defined as vancomycin and/or teicoplanin MICs ≥ 8 mg/L and 12 mg/L, respectively [19].

#### 5.4.3. Brain–Heart Infusion–Casein–Vancomycin Plates

Brain–heart infusion (BHI) agar plates (Difco, Becton Dickinson and Company) supplemented with 16 g/L pancreatic digest of casein (Becton Dickinson) and varying concentrations of vancomycin (Sigma Chemical Company, St. Louis, MO, USA) were used: 3 mg/L (BHI-3) and 4 mg/L (BHI-4).

These BHI–casein–vancomycin agar plates were inoculated with 4 separate 10 µL drops of 0.5 McFarland standard suspension per isolate. After drying for 5 min, plates were incubated at 35 °C at air. Bacterial growth was visually inspected at 24 and 48 h, and the number of the visible colonies in each drop was counted. A strain was considered hVISA if at least one drop showed two or more colonies after incubation; a droplet with a count of 20 or more CFU was considered as confluent growth [19].

### 5.5. Study of PAP-AUC

A 0.5 McFarland standard suspension of each studied strain was prepared from an overnight culture in TSB broth (Becton Dickinson and Company). Ten-fold serial dilutions in sterile saline were performed down 10^−6^ dilution. Ten microliters of each dilution were inoculated in BHI plates containing varying vancomycin concentrations: 0, 0.25, 0.50, 1.0, 1.5, 2.0, 3, 4 and 6 mg/L.

Plates were air dried and incubated at 35 °C, and viable counts were completed at 24 and 48 h. The log 10 number of CFU/mL for each strain was plotted as a function of vancomycin concentrations, and the area under the curve (AUC) was calculated using the GraphPad Prism (San Diego, CA, USA). Isolates were considered hVISA if the AUC ratio of the strain tested versus the Mu ATCC 700698 was greater than 0.9 [19].

### 5.6. Analysis of Cell Wall Thickening

Transmission electron microscopy (Joel model JEM 10–10) was used to visualize cellwall thickening in a vancomycin-decreased susceptibility strain. Bacteria were exposure to sub-inhibitor concentrations of vancomycin and then analyzed during the exponential growth phase. As a control, ATCC 25923 and VISA Mu ATCC 700699 strains were also grown to the exponential phase. The bacteria were harvested, washed, and fixed with a glutaraldehyde/formalin (2.5%/10%) solution in 0.1 M phosphate-buffered saline (PBS), pH 6.0. After that, the bacteria were post-fixed with osmium tetroxide, contrasted with uranyl acetate, and dehydrated in graded concentrations of ethyl alcohol (20, 30, 40, 50, 60, 70, 80, 90, and 100%). Transverse thin sections from samples embedded in resin were mounted on grids, which was followed by treatment with lead citrate. The samples were stained with 1% phosphotungstic acid at pH 7.2 and visualized by transmission electron microscopy. Observation and acquisition was performed using a transmission electron microscope operated at 100 kV and equipped with a Hamamatsu C4742-95 digital camera. The images were processed at 100,000× and analyzed to determine the cellwall thickness from an average of 10 cells per bacterial strain [30].

### 5.7. Statistical Analysis

Qualitative variables were described using summary measures (frequencies). The MIC_90_ for vancomycin, defined as the lowest concentration of antimicrobial needed to inhibit bacterial growth of 90% of analyzed strains, was determined. For comparison of cellwall thickness, the ANOVA test was performed after confirming the normal distribution and homogeneity of the variance. A post hoc test for independent samples was used, and a *p* value < 0.05 was considered statistically significant.

## 6. Conclusions

In this MRSA set recovered in a pediatric reference hospital, there were no detected VISA or VRSA strains despite a documented increase in the use of vancomycin since 2002. This finding suggests that these resistance mechanisms are not currently a significant burden at the CHPR. However, continuous laboratory surveillance remains crucial, particularly for children with comorbidities and extended hospital stays and who receive vancomicyn.

This study identified only one hVISA strain, which was not detected by any of the currently recommended screening methods employed. This highlights the limitations of these methods for identifying strains with decreased susceptibility to glycopeptides (DSG) and stand out the need for robust, simple, and clinically applicable procedures for DSG detection. Implementing such methods in clinical microbiology laboratories would enable a more precise evaluation of the potential role of hVISA strains in vancomycin treatment failures.

The BHI4 plate inoculated with 0.5 McFarland suspension, 48 h of incubation and a single colony per drop interpreted as a positive result successfully detected the only hVISA strain confirmed by PAP-AUC. However, further evaluation is necessary to assess effectiveness for screening hVISA in clinical settings.

The identified hVISA strain exhibited an increased cell wall thickness, belonged to ST30 with SCC*mec* type IV, and carried the *lukS-F* gene, sharing similar genetic characteristics with a previously identified hVISA strain in neighboring Brazil. Comparative analysis of these isolates could be valuable in understanding the potential regional dissemination of hVISA strains.

Further studies are necessary to recover, characterize, and compare hVISA strains, particularly in the context of severe staphylococcal infections affecting both children and adults.

## Figures and Tables

**Table 1:** Results of the screening methods and the population profile and area under the curve (PAP-AUC) experiments in the 15 selected methicillin-resistant *S. aureus* isolated in Uruguay 2009–2019.

Strain Number	AUC Strain	AUC Strain/AUCμ3	VITEK^®^ VA TE		*E*-Test VA TE		Macro *E*-Test VA TE		BHI4_24_ VA (CFU)	BHI4_48_ VA (CFU)
1	16.05	0.799	1	0.5	1	0.5	3	3	1	1
2	16.3	0.811	0.5	0.5	1	0.75	3	3	1	4
3	17.55	0.873	0.5	0.5	1	1.5	1	2	0	0
4	15.25	0.759	0.5	0.5	1	0.25	1.5	1.5	0	1
5	13.98	0.685	1	0.5	1	0.75	1.5	1.5	0	1
6	16.72	0.832	1	0.5	1	0.75	2	2	0	1
**7**	**19.01**	**0.946**	**1**	**0.5**	**1**	**0.75**	**2**	**1.5**	**0**	**1**
8	14.48	0.720	1	0.5	1	1	2	3	1	1
9	17.15	0.853	1	0.5	1.5	0.38	2	3	0	0
10	9.83	0.489	0.5	0.5	1	0.75	3	2	1	2
11	16.48	0.822	1	0.5	1.5	1	2	2	0	0
12	16.01	0.797	1	0.5	1.5	0.5	2	2	0	0
13	9.46	0.471	0.5	0.5	1	0.5	3	3	1	1
14	16.4	0.816	1	0.5	0.75	1	2	3	2	2
15	17.55	0.873	1	0.5	1	0.75	1.5	2	1	1
μ3	20.09	1	ND	ND	ND	ND	ND	ND	ND	ND

CFU: colony-forming unit, AUC: area under the curve, BHI4_24_: brain–heart infusion at 24 h, BHI4_48_: brain–heart infusion at 48 h, VA: vancomycin, TE: teicoplanin. ND, not done. The strain defined as hVISA is shown in bold.

**Table 2:** Microbiological characteristics, infection source and results of glycopeptide resistance screening methods of the 15 selected strains from 52 methicillin-resistant *S. aureus* isolated in Uruguay 2009–2019.

Strain Number	AUC Strain/AUCμ3	Source	*lukS-F* Gene	SCC*mec* Type	Resistance Phenotype	Sequence Type /Clonal Complex
1	0.799	bacteremia	−	II	OX, CIP, cMLSb	ST-868/CC-5
2	0.811	adenophlegmon	+	IV	OX	ST-8/CC-8
3	0.873	arthritis	−	IV	OX	ND
4	0.759	bacteremia	−	II	OX, CIP, cMLSb	ST-5/CC-5
5	0.685	bacteremia	−	IV	OX, iMLSb	ST-30/CC-30
6	0.832	bacteremia	+	IV	OX	ND
**7**	**0.946**	**bacteremia**	**+**	**IV**	**OX**	**ST-30/CC-30**
8	0.720	pneumonia	−	II	OX, CIP, cMLSb, STX, GN	ST-5/CC-5
9	0.853	arthritis	−	V	OX	ST-8/CC-8
10	0.489	osteomyelitis	+	IV	OX, iMLSb	ND
11	0.822	endocarditis	−	IV	OX	ST-30/CC-30
12	0.797	osteomyelitis	+	II	OX	ST-577/CC-121
13	0.471	arthritis	+	IV	OX	ND
14	0.816	arthritis	−	V	OX	ND
15	0.873	pneumonia	−	IV	OX, iMLSb	ST-434/CC-30

AUC (area under the curve), SCC*mec* (Staphylococcal chromosomal cassette *mec*), *lukS-F* (Panton–Valentine leukocidin), iMLSb: inducible macrolides, lincosamides, and type B streptogramin, cMLSb: constitutive macrolides, lincosamides, and type B streptogramin, CIP (ciprofloxacin), OX (oxacillin), GN (gentamicin), STX (trimethoprim–sulfamethoxazole). ST: sequence type, CC: clonal complex. The strain defined as hVISA is shown in bold. ND, not done. +, positive; −, negative

**Table 3:** Primers used in the multiplex PCR to determine SCC*mec* type of 52 methicillin-resistant *S. aureus* isolated in Uruguay 2009–2019 [25].

Primer Name	SCC*mec* Amplicon	Oligonucleotide Sequence (5′-3′)	Amplicon Size
mI-1	*mec* complex A	AATGGCGAAAAAGCACAACA	480 bp
mI-2	*mec* complex A	GACTTGATTGTTTCCTCTGTT	
mcR-2	*mec* complex A	CGCTCAGAAATTTGTTGTGC	1597 bp
mcR-3	*mec* complex A	ATCTCCACGTTAATTCCATT	
IS1272	*mec* complex B	ATTTTGGGTTTCACTCGGAT	565 bp
mecR1	*mec* complex B	CAAATATTAAAGAACGTGTT	
mA7	*mec* complex C	ATATACCAAACCCGACAACTACA	804 bp
iS2	*mec* complex C	TGAGGTTATTCAGATATTTCGATGT	
ccr-β2	*ccr*AB1	ATTGCCTTGATAATAGCCITCT	700 bp
ccr-α2	*ccr*AB1	AACCTATATCATCAATCAGTACGT	
ccr-β2	*ccr*AB2	ATTGCCTTGATAATAGCCITCT	1000 bp
ccr-α3	*ccr*AB2	TAAAGGCATCAATGCACAAACACT	
ccr-β2	*ccr*AB3	ATTGCCTTGATAATAGCCITCT	1600 bp
ccr-α4	*ccr*AB3	GCTCAAAAGCAAGCAATAGAAT	
ccrC-F2	*ccr*C	GTACTCGTTACAATGTTTGG	449 bp
ccrC-R2	*ccr*C	ATAATGGCTTCATGCTTACC	

**Table 4:** Schemes to determine SCC*mec* type according to *ccr* complex and *mec* complex in 52 methicillin-resistant *S. aureus* isolated in Uruguay 2009–2019. Adapted from International Working Group on the Staphylococcal Cassette Chromosome elements [26].

SCC*mec*Type	*Ccr*Complex	*Mec*Complex
I	A1B1	B
II	A2B2	A
III	A3B3	A
IV	A2B2	B
V	C1	C2
VI	A4B4	B
VII	C1	C1
VIII	A4B4	A
IX	A1B1	C2
X	A1B6	C1
XI	A1B3	E

## Data Availability

All data from this research are available in the text.

## References

[1] McGuinness W.A., Malachowa N., De Leo F.R. (2017). Vancomycin Resistance in *Staphylococcus aureus*. Yale J. Biol. Med..

[2] Hiramatsu K. (2001). Vancomycin-Resistant *Staphylococcus aureus*: A new Model of Antibiotic Resistance. Lancet Infect. Dis..

[3] Tenover F.C., Moellering R.C. (2007). The Rationale for Revising the Clinical and Laboratory Standards Institute Vancomycin Minimal Inhibitory Concentration Interpretive Criteria for *Staphylococcus aureus*. Clin. Infect. Dis..

[4] Kim T., Kim E.S., Park S.Y., Sung H., Kim M.-N., Kim S.-H., Lee S.-O., Choi S.-H., Jeong J.-Y., Woo J.H. (2017). Phenotypic Changes of Methicillin-Resistant *Staphylococcus aureus* during Vancomycin Therapy for Persistent Bacteraemia and Related Clinical Outcome. Eur. J. Clin. Microbiol. Infect. Dis..

[5] Hu Q., Peng H., Rao X. (2016). Molecular Events for Promotion of Vancomycin Resistance in Vancomycin Intermediate *Staphylococcus aureus*. Front. Microbiol..

[6] Castro B.E., Rios R., Carvajal L.P., Vargas M.L., Cala M.P., León L., Hanson B., Dinh A.Q., Ortega-Recalde O., Seas C. (2022). Multiomics Characterization of Methicillin-Resistant *Staphylococcus aureus* (MRSA) Isolates with Heterogeneous Intermediate Resistance to Vancomycin (hVISA) in Latin America. J. Antimicrob. Chemother..

[7] Shariati A., Dadashi M., Moghadam M.T., van Belkum A., Yaslianifard S., Darban-Sarokhalil D. (2020). Global Prevalence and Distribution of Vancomycin Resistant, Vancomycin Intermediate and Heterogeneously Vancomycin Intermediate *Staphylococcus aureus* Clinical Isolates: A Systematic Review and Meta-Analysis. Sci. Rep..

[8] Telechea H., Speranza N., Lucas L., Santurio A., Giachetto G., Algorta G., Nanni L., Pírez M.C. (2009). Antibiotic consumption and antimicrobial susceptibility evolution in the Centro Hospitalario Pereira Rossell in methicillin resistant *Staphylococcus aureus* era. Rev. Chil. Infectol..

[9] Di Gregorio S., Perazzi B., Ordoñez A.M., DeGregorio S., Foccoli M., Lasala M.B., García S., Vay C., Famiglietti A., Mollerach M. (2015). Clinical, Microbiological, and Genetic Characteristics of Heteroresistant Vancomycin-Intermediate *Staphylococcus aureus* Bacteremiaina Teaching Hospital. Microb. Drug Resist..

[10] Chamon R.C., Marques L.M., Timenetsky J., da Costa Rachid C.T.C., Ferreira R.B.R., de Oliveira T.L.R., Glatthardt T., de Oliveira Moreira L., dos Santos K.R.N. (2020). Genome Sequence of a Highly Virulent Pvl-Positive Vancomycin-Intermediate-Resistant *Staphylococcus aureus* Sequence Type 30. Curr. Genom..

[11] Sola C., Lamberghini R.O., Ciarlantini M., Egea A.L., Gonzalez P., Diaz E.G., Huerta V., Gonzalez J., Corso A., Vilaro M. (2011). Heterogeneous Vancomycin-Intermediate Susceptibility in a Community-Associated Methicillin-Resistant *Staphylococcus aureus* Epidemic Clone, in a Case of Infective Endocarditis in Argentina. Ann. Clin. Microbiol. Antimicrob..

[12] Di Gregorio S., Vielma J., Haim M.S., Rago L., Campos J., Kekre M., Abrudan M., Famiglietti Á., Canigia L.F., Rubinstein G. (2023). Genomic Epidemiology of *Staphylococcus aureus* Isolated from Bloodstream Infections in South America during 2019 Supports Regional Surveillance. Microb. Genom..

[13] Pardo L., Vola M., Macedo-Viñas M., Machado V., Cuello D., Mollerach M., Castro M., Pírez C., Varela G., Algorta G. (2013). Community-Associated Methicillin-Resistant *Staphylococcus aureus* in Children Treated in Uruguay. J. Infect. Dev. Ctries..

[14] Ma X.X., Galiana A., Pedreira W., Mowszowicz M., Christophersen I., Machiavello S., Lope L., Benaderet S., Buela F., Vicentino W. (2005). Community-Acquired Methicillin-Resistant *Staphylococcus aureus*, Uruguay. Emerg. Infect. Dis..

[15] Kim Y.-H., Park J., Chung H.-S. (2023). Genetic Characterization of Tetracycline-Resistant *Staphylococcus aureus* with Reduced Vancomycin Susceptibility Using Whole-Genome Sequencing. Arch. Microbiol..

[16] Howden B.P., Peleg A.Y., Stinear T.P. (2014). The Evolution of Vancomycin Intermediate *Staphylococcus aureus* (VISA) and Heterogenous-VISA. Infect. Genet. Evol..

[17] Richter S.S., Satola S.W., Crispell E.K., Heilmann K.P., Dohrn C.L., Riahi F., Costello A.J., Diekema D.J., Doern G.V. (2011). Detection of *Staphylococcus aureus* Isolates with Heterogeneous Intermediate-Level Resistance to Vancomycin in the United States. J. Clin. Microbiol..

[18] Khatib R., Riederer K., Sharma M., Shemes S., Iyer S.P., Szpunar S. (2015). Screening for Intermediately Vancomycin-Susceptible and Vancomycin-Heteroresistant *Staphylococcus aureus* by Use of Vancomycin-Supplemented Brain Heart Infusion Agar Biplates: Defining Growth Interpretation Criteria Based on Gold Standard Confirmation. J. Clin. Microbiol..

[19] Satola S.W., Farley M.M., Anderson K.F., Patel J.B. (2020). Comparison of Detection Methods for Heteroresistant Vancomycin-Intermediate *Staphylococcus aureus*, with the Population Analysis Profile Method as the Reference Method. J. Clin. Microbiol..

[20] Katayama Y., Azechi T., Miyazaki M., Takata T., Sekine M., Matsui H., Hanaki H., Yahara K., Sasano H., Asakura K. (2017). Prevalence of Slow-Growth Vancomycin Non susceptibility in Methicillin-Resistant *Staphylococcus aureus*. Antimicrob. Agents Chemother..

[21] Wootton M., Howe R.A., Hillman R., Walsh T.R., Bennett P.M., MacGowan A.P. (2001). A Modified Population Analysis Profile (PAP) Method to Detect Hetero-Resistance to Vancomycin in *Staphylococcus aureus* in a UK Hospital. J. Antimicrob. Chemother..

[22] Investigation and Control of Vancomycin-Resistant *Staphylococcus aureus* (VRSA): 2015 Update. https://www.cdc.gov/hai/pdfs/vrsa-investigation-guide-05_12_2015.pdf.

[23] Silveira A.C.d.O., Sambrano G.E., Paim T.G.d.S., Caierão J., de Cordova C.M.M., d’Azevedo P.A. (2014). Is Pre diffusion Test an Alternative to Improve Accuracy in Screening hVISA Strains and to Detect Susceptibility to Glycopeptides/Lipopeptides?. Diagn. Microbiol. Infect. Dis..

[24] Oliveira D.C., de Lencastre H. (2002). Multiplex PCR Strategy for Rapid Identification of Structural Types and Variants of the Mec Element in Methicillin-Resistant *Staphylococcus aureus*. Antimicrob. Agents Chemother..

[25] Kondo Y., Ito T., Ma X.X., Watanabe S., Kreiswirth B.N., Etienne J., Hiramatsu K. (2007). Combination of Multiplex PCRs for Staphylococcal Cassette Chromosome *mec* Type Assignment: Rapid Identification System for Mec, Ccr, and Major Differences in Junkyard Regions. Antimicrob. Agents Chemother..

[26] SCCmec International Working Group on the Staphylococcal Cassette Cromosome Elements (IWG-SCC). https://www.sccmec.org/index.php/en/.

[27] Lina G., Piémont Y., Godail-Gamot F., Bes M., Peter M.-O., Gauduchon V., Vandenesch F., Etienne J. (1999). Involvement of Panton-Valentine Leukocidin—Producing *Staphylococcus aureus* in Primary Skin Infections and Pneumonia. Clin. Infect. Dis..

[28] Enright M.C., Day N.P.J., Davies C.E., Peacock S.J., Spratt B.G. (2000). Multilocus Sequence Typing for Characterization of Methicillin-Resistant and Methicillin-Susceptible Clones of *Staphylococcus aureus*. J. Clin. Microbiol..

[29] Clinical and Laboratory Standards Institute (CLSI) (2023). Performance Standards for Antimicrobial Susceptibility Testing.

[30] Cázares-Domínguez V., Cruz-Córdova A., Ochoa S.A., Escalona G., Arellano-Galindo J., Rodríguez-Leviz A., Hernández-Castro R., López-Villegas E.O., Xicohtencatl-Cortes J. (2015). Vancomycin Tolerant, Methicillin-Resistant *Staphylococcus aureus* Reveals the Effects of Vancomycin on Cell Wall Thickening. PLoS ONE.

